# Effects of multiple stressors on the behavior of fish in the presence and absence of a predator

**DOI:** 10.1093/beheco/arag083

**Published:** 2026-07-29

**Authors:** Amelia Munson, Daphne Cortese, Izzy C Tiddy, Helena Norman, Shaun S Killen

**Affiliations:** School of Biodiversity, One Health and Veterinary Medicine, College of Biomedical and Life Sciences, University of Glasgow, University Avenue, Glasgow, G128QQ, United Kingdom; Department of Wildlife, Fish and Environmental Studies, Swedish University of Agricultural Sciences, Umeå, 90183, Sweden; School of Biodiversity, One Health and Veterinary Medicine, College of Biomedical and Life Sciences, University of Glasgow, University Avenue, Glasgow, G128QQ, United Kingdom; Department of Fish Biology, Fisheries and Aquaculture, IGB, Berlin, 12587, Germany; School of Biodiversity, One Health and Veterinary Medicine, College of Biomedical and Life Sciences, University of Glasgow, University Avenue, Glasgow, G128QQ, United Kingdom; School of Biodiversity, One Health and Veterinary Medicine, College of Biomedical and Life Sciences, University of Glasgow, University Avenue, Glasgow, G128QQ, United Kingdom; School of Biodiversity, One Health and Veterinary Medicine, College of Biomedical and Life Sciences, University of Glasgow, University Avenue, Glasgow, G128QQ, United Kingdom

**Keywords:** temperature, hypoxia, social behavior, activity, sticklebacks, minnows, multispecies

## Abstract

Temperature and oxygen concentration can alter the behavior of animals. While often studied in isolation, these factors frequently co-occur. Together they may have nonadditive effects. The effect of abiotic stressors may also change in the presence of biotic stressors, like predators, due to altered costs and benefits of behaviors. Additionally, species may balance the demands of co-occurring stressors differently depending on their unique needs. Here, we investigated the effects of acute exposures to elevated temperature (16 °C versus 12 °C) and decreased dissolved oxygen (30% versus 100% DO) on the social behavior and activity of two co-occurring freshwater fish (common minnow, *Phoxinus phoxinus*, and three-spined stickleback, *Gasterosteus aculeatus*) before and after a simulated predator attack. Prior to the release of the model predator, the effect of one stressor was dependent on the other stressor in minnows. Evidence suggested that minnows were more social in warmer waters, but only in hypoxic conditions, and were less active under hypoxia, but only in warm water. Sticklebacks were more active in warm water at both oxygen concentrations. Following predator release, there were no differences in social behavior, likely because of overall reductions in activity. Our study highlights the need to consider stressor combinations. Minnows appeared to compensate for one abiotic stressor such that effects were only apparent when the stressors were experienced together. However, the arrival of the “predator” masked differences between the treatment groups. Different responses between the species suggest that studies in species interactions are needed to understand community level impacts of multiple stressors.

## Teaser

When acutely exposed to elevated temperature and reduced oxygen concentration, minnows and stickleback fish altered their behavior in different ways. Minnows only changed their behavior when experiencing both stressors in combination, but stickleback was more active in warm water, regardless of the oxygen concentration. Predator presence reduced movement in both species, masking differences in behavior. If co-occurring species differ in their responses to the same environmental factors, it may impact community dynamics.

## Introduction

Environmental factors are highly dynamic, rarely changing in isolation. Indeed, changes in some factors are mechanistically linked to changes in others, increasing the likelihood of their co-occurrence. For example, in aquatic ecosystems, hypoxia can occur when natural or anthropogenic increases in nutrient availability lead to eutrophication and proliferation of oxygen-consuming phytoplankton and bacteria (Breitburg et al. 2018). Warm water can exacerbate the resulting decrease in dissolved oxygen (DO) because phytoplankton have an increased metabolic demand for oxygen at higher temperatures (McBryan et al. 2013). Additionally, as water temperature increases, solubility of oxygen decreases, which can further amplify hypoxic conditions (Altieri and Gedan 2015). Factors that are likely to change together should be studied together, because the consequences of exposure to multiple stressors may differ to that of exposure to each factor in isolation.

The effects of multiple stressors can be classified as either additive, antagonistic or synergistic (Todgham and Stillman 2013). The potential for synergisms, where the effect of one factor depends on the presence of the other, means that predicting the effect of exposure to simultaneous changes from single-factor studies is challenging. For example, when experienced on their own, changes to both temperature and DO can influence the ability and motivation of animals to engage in a suite of behaviors. Changes in temperature can alter behaviors (eg activity (Abram et al. 2017) and foraging (Killen et al. 2024a)) because in ectotherms like fish, metabolic needs tend to increase over a limited temperature range (Fry 1971); changes in metabolism may then alter the benefits of different behaviors (Killen et al. 2021). Conversely, aerobic metabolic processes require oxygen, so strongly hypoxic conditions can limit the capacity of an individual to engage in aerobic respiration; this can lead to reductions in activity to lower metabolic demands (eg Domenici et al. 2007) or temporary increases in activity to move away from hypoxic areas (Killen et al. 2012; Domenici et al. 2013). Despite these clear effects of single stressors, predicting the response to simultaneous changes in the two factors is challenging. There are both theoretical justifications and (limited) empirical evidence that when experienced together, elevated temperature and hypoxia can have synergistic effects on a variety of physiological and fitness-related metrics in fish (reviewed in Claireaux et al. 2000; McBryan et al. 2013). For example, hypoxia tolerance is reduced at elevated temperatures, but the effect is species-specific (He et al. 2015). Whether these physiological effects translate to changes in behavior though, is poorly understood (Lopez et al. 2023; Munson et al. 2024). Moreover, most studies that do look at behavioral effects focus solely on the influence of changes to environmental conditions on patterns of activity, which, in controlled lab studies, is almost always measured with isolated individuals. Changes in the environment may also impact an individual's motivation to spend time in a group, which could have impacts on fitness-related traits.

For many animals, including most species of fish, social behavior and grouping is a key trait that is the result of balancing many costs and benefits (Ward and Webster 2016). Staying in a group can, for example, reduce the risk of individual predation threat (Lima 1995; Roberts 1996; Ioannou et al. 2012). Individuals in groups may also have higher reproductive success (Canestrari et al. 2008) and an increased likelihood of finding food (Pitcher et al. 1982). However, group living can increase disease transfer (Nunn et al. 2015) and competition for resources (Ward and Webster 2016). Importantly, the costs and benefits of grouping can vary as a function of the environment. For example, juvenile walleye pollock, *Theragra chalcogramma*, increase cohesion in response to an acute predator threat but decrease cohesion with decreased food availability (Sogard and Olla 1997).

Independently, both temperature and oxygen concentration can affect social behavior in fish. In three-spined sticklebacks, *Gasterosteus aculeatus*, for instance, warmer environments can reduce sociability (Pilakouta et al. 2023), potentially due to changes in metabolism that may shift the benefits of remaining in a group (Killen et al 2017). Hypoxic conditions can also reduce cohesion in schooling fish, if individuals move away from each other to reduce competition for limited oxygen (Domenici et al. 2017). However, studies that investigated the independent effects of temperature and DO on social behavior have produced mixed results and there is limited work on individual sociability and group cohesion when these stressors co-occur (Tiddy et al. 2024). Determining *why* factors like temperature and hypoxia alter behavior is even more challenging. If the environment changes, so too might the costs and benefits associated with being social, so an individual's motivation to be social may change. Alternatively, it may be more challenging to maintain cohesion under certain environmental condition (eg, due to changes in cognitive ability (Munson et al. 2025) or sensory perception (Robinson et al. 2013; Ehlman et al. 2019)), so even if an animal is motivated to be social, it may lack the capacity. In some cases, individuals may alter other behaviors to maintain sociability in changed environments. For example, striped surfperch, *Embiotoca lateralis,* maintained shoal cohesion at very low levels of oxygen availability (15% air saturation) by reducing their swim speed and turning rate (Cook et al. 2014). However, if animals are less social under certain contexts, distinguishing between changes to motivation or capacity is challenging. Studies that directly manipulate factors associated with the costs and benefits of social behavior may be able to better untangle why social behaviors change across different environmental conditions.

Predicting responses to multiple stressors across species is further complicated by the fact that not all species are equally affected by a given change in an environmental condition. Different species may also respond to similar challenges with different strategies. For example, some fish species are less cohesive at higher temperatures (eg Colchen et al. 2017), whereas others are more cohesive (eg Allibhai et al. 2023). Species also differ in their tolerance for different factors, both in terms of optimum and breadth. For example, even fish that live in similar niches and are acclimated to the same temperature, can exhibit different critical thermal maximums (Radtke et al. 2022). Thus, a change that may be considered stressful to one species may not be to another. However, because individual species can be influenced by the actions of other species, a change in a factor may have overall detrimental effects to a species even if it is within its tolerance range, if the change impacts a different species with which the focal species interacts. For example, differences in thermal performance between the predatory largemouth bass, *Micropterus salmoides*, and juvenile Chinook salmon, *Oncorhynchus tshawytscha*, are likely to lead to increased consumption of salmon in warmer waters (McInturf et al. 2022). Similar changes to species interactions are also possible for other types of relationships, including species that benefit from co-occurring. Single studies though rarely compare behavioral responses to environmental change in different, co-occurring species, so species comparison can be challenging, particularly if procedures are not similar across experiments.

In this study, we explored the direct and interactive effects of acute exposure to increased temperature and hypoxia on individual social behavior and activity in fish. We measured both behaviors before and after a simulated predator threat to determine if changes to the costs and benefits associated with shoaling impacted behavior. We compared responses in common minnows, *Phoxinus phoxinus*, and three-spined sticklebacks, *G. aculeatus*, two freshwater fish species that often coexist. Both species display schooling behavior, are exposed to similar predators, and naturally experience seasonal and daily variation in temperature, as well as variation in DO, making them excellent study species for this experiment. The changes in temperature and oxygen concentration used here likely do not represent the same level of stress to each species, but that was intentional as we were interested more in how the same environmental change impacted two different species that co-occur in the same environment, not on the impact of stress per se. We predicted that both species would increase activity and decrease social behavior in warm water due to changes in metabolic activity and altered benefits and costs of remaining social (Killen et al. 2017). We predicted that both species would decrease activity and social behavior in hypoxic water to conserve oxygen and decrease competition. We predicted greater reductions in social behavior when water was both warm and hypoxic. If reductions in social behavior are a result of altered costs and benefits, we predicted that a simulated predator attack would lead to an increase in social behavior. However, if temperature and oxygen constrain individuals such that they are no longer able to be as social (ie, changes interfere with cognitive function or perception) then a simulated predator attack should not influence social behavior.

## Materials and methods

### Animal collection and husbandry

Wild three-spined sticklebacks, *Gasterosteus aceleatus*, and common minnow, *Phoxinus phoxinus*, were collected from the River Kelvin (Glasgow 55° 52′ 42″ N 004° 17′ 03″ W) in June 2023 using dip-nets and minnow traps. Following collection, fish were immediately brought to the lab at the University of Glasgow and placed in 2 stock tanks (42 L each), separated by species (*n* = 76 sticklebacks and *n* = 73 minnows). Both tanks had gravel and artificial plants for enrichment. Water in the system was recirculating and UV sterilized. Fish were acclimated to laboratory conditions (12 °C, 12L:12D photoperiod) for at least 1 week. Individuals of both species that took on breeding colors were removed from the stock tanks and euthanized without being used in the experiment (*n* = 2 sticklebacks, 1 minnow). Prior to the start of behavior assays, 10 fish of each species of average length were haphazardly removed from the stock tanks and moved to 2 separate 27 L tanks (separated by species). These tanks were maintained under the same conditions as stock tanks. These fish were later used as stimulus shoals for the experiments. All fish were fed bloodworms twice a day for the duration of the acclimation period and the experiment, except for the day that an individual was used in behavior testing.

### Experimental procedure

The behavior (time spent moving and time spent with a group) of individual fish was recorded before and after exposure to a model predator stimulus. Behavior was measured in an arena in which fish could be acutely exposed to stressors. Fish of both species were haphazardly selected for 1 of 4 combinations of acute changes in oxygen availability (dissolved oxygen—DO) and temperature: control—100% DO and 12 °C (*N* = 18, sticklebacks, 15, minnows); elevated temperature—100% DO and 16 °C (*N* = 14, sticklebacks, 16, minnows); decreased oxygen availability—30% DO and 12 °C (*N* = 18, sticklebacks, 15, minnows); and both elevated temperature and decreased oxygen availability—30% DO and 16 °C (*N* = 14, sticklebacks, 16, minnows). The sample sizes were uneven across conditions because extra fish were run in some conditions when the model predator did not release properly. In trials when the predator did not release correctly, behavior measures were still calculated for pre-predator trials, but these fish were omitted from post-predator exposure analysis. Fish in all treatments experienced the same general procedure detailed below.

The magnitudes of change in oxygen concentration and temperature were selected to be within the range recorded in the river where the fish were captured. This was done because we were interested in the effects of an acute change that could shift behavior without being near their thermal maxima. Therefore, although temperatures of 16 °C may not be stressful per se for either species, a rapid change over a short time scale (in our case 4 °C in 40 min) could elicit stress and behavioral responses even at temperatures that these species regularly experience in summer months (Alfonso et al. 2021). The time course of exposure was chosen to mimic acute exposure in the wild as animals move through thermal or oxygen gradients. Both the level and time course of exposure have the potential to alter effects, and we choose conditions that are more likely to alter behavior before physiological acclimation can occur. For example, the time course of hypoxia exposure has been shown to affect whether there is an impact to schooling in anchovies (Moss and McFarland 1970). Thus, in our study we choose to modify oxygen from 100% to 30% over 10 min. Dissolved oxygen was controlled and expressed as percentage air saturation rather than as an absolute concentration (mg O_2_ L^−1^). Because oxygen solubility declines with increasing temperature, this means that absolute oxygen concentration differed between temperatures for a given % saturation. However, holding mg O_2_ L^−1^ constant across temperatures would result in different oxygen partial pressures and degrees of hypoxia relative to saturation, such that individuals at higher temperature would experience comparatively higher oxygen availability. Expressing oxygen as % air saturation therefore ensured that treatments represented comparable hypoxic conditions relative to equilibrium at each temperature, while reflecting the natural co-variation between warming and oxygen availability in aquatic systems.

The evening before each day of behavioral testing (at 18:00), 6 minnows and 6 sticklebacks were haphazardly selected from stock tanks and moved to 2 smaller acclimation tanks (27 L, 12 °C, 100% DO), separated by species, in the same recirculating system and housed overnight. Each tank had gravel (mixed “natural” color—brown, white, gray, 2 cm depth, mixed sizes) and 3 small (∼10 cm) artificial plants for enrichment (Jones et al. 2021). On a given day, all fish were tested at the same temperature (12 °C or 16 °C) so that the temperature of the testing room and water could be adjusted accordingly to maintain water temperature in the arenas. Two trials were run at the same time in 2 identical arena set-ups, 1 at hypoxia (30% DO) and 1 at normoxia (100% DO). On a given trial day, fish were tested in species blocks (ie 2 sets (1 for each species) of 3 trials, each trial consisting of 2 individuals of the same species tested in separate arenas). The order of species tested was counterbalanced between days. Temperature of the trials was alternated every 2 d (so that the same species was not always tested first for a given temperature). On elevated temperature days, 40 min before the start of the first trial for a given species, the temperature of the tank was gradually increased from 12 to 16 °C using an aquarium heater, increasing the temperature roughly 1 degree every 10 min. Trial number was included in all future analyses to account for the fact that fish had been exposed to the temperature for slightly different lengths of time. All trials were run between 0900 and 1700 h.

**Figure 1:**
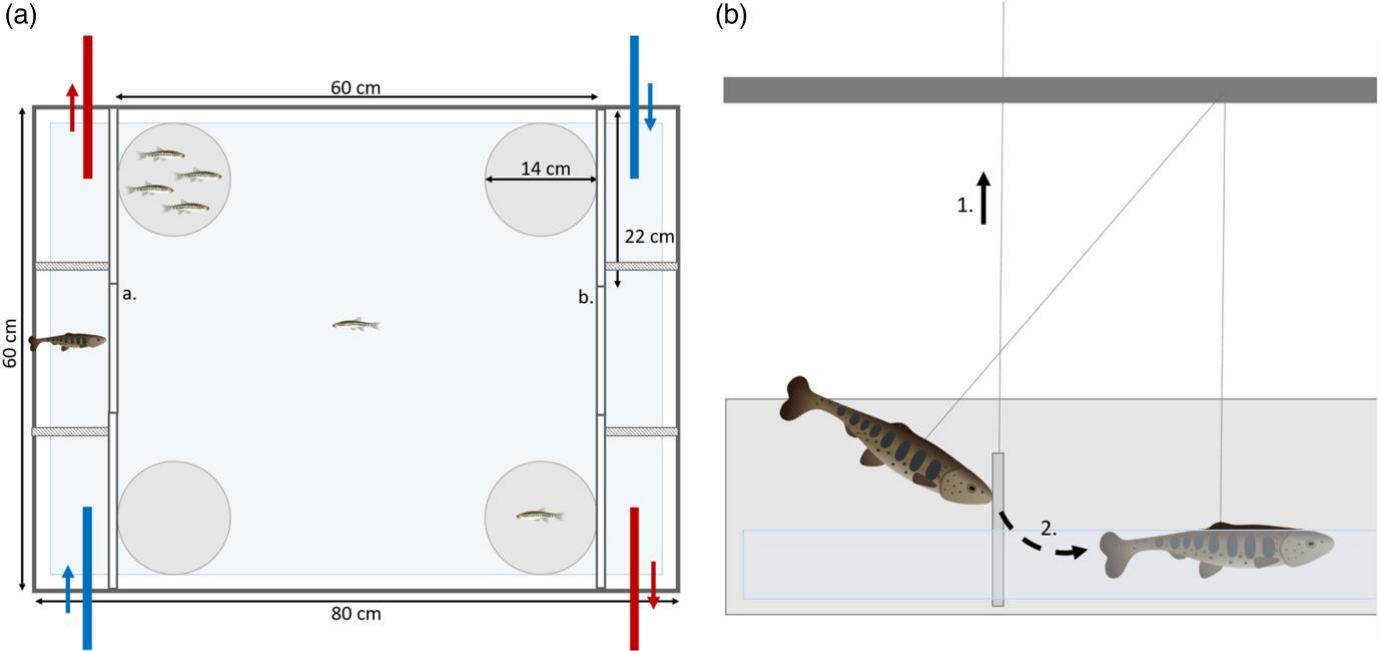
a) Arena overview. Grey circles in four corners indicate glass jars. The large shoal and small shoal were always diagonally across from each other but rotated every trial. Solid white rectangles indicate walls and removable doors. Behind one door a.a) was a model predator that would swing forward on fishing line when the door was removed. Both doors (a.a. and a.b) were removed on every trial. Striped rectangles indicate mesh. Blue and red rectangles leading out of the arena indicate tubing; water was pumped out of the tank (red arrows, upper left and lower right), through a recirculating system to obtain appropriate oxygen concentration, and then returned to the tank (blue arrows, upper right and lower left). b) Model predator release system (not drawn to scale). (1) Doors were removed by an experimenter behind a white curtain pulling the attached fishing line. (2) The model predator then swung forward to rest in the middle of the tank, equidistant from all shoal containers.

For each trial, 2 fish from the acclimation tanks were netted and transferred in a bucket to the experimental room where they were gently placed individually in a plastic tube with small holes in it in the middle of an experimental arena with water maintained at the test temperature. Each experimental arena was a large rectangular plastic tank (80 × 60 × 22 cm; water depth: 4 cm) with plastic walls creating an internal compartment for fish to move around in (60 × 60 × 22 cm) (Fig. 1a). The perimeter walls were covered with waterproof white paper to facilitate automated tracking and the whole arena was surrounded by white curtains to diffuse light and reduce disturbances. In each corner of the center chamber, there was a cylindrical glass container (diameter: 14 cm, 32 cm between containers). Four fish were placed in 1 container to serve as the large stimulus shoal. One fish was placed in the container diagonally across from the large shoal to serve as a small stimulus shoal. The size of the shoal container was chosen to limit movement of the stimulus shoals, to reduce the degree to which differences in shoal behavior could account for differences in the focal individual's movement. The remaining 2 containers were empty controls. We used this design, as opposed to a dichotomous choice of multiple versus no fish, because both species are highly social. Giving fish the option between a large shoal, small shoal and no shoal can reveal variation in sociability between individuals that is masked in a dichotomous choice test (Wark et al. 2011). Distance between stimulus containers can be important when measuring social tendency (McInnes et al. 2025; Swaney et al. 2025). The size of our tank was chosen as a compromise between being small enough that we could reasonably control temperature and oxygen concentration while being large enough to measure social tendency: it has previously been shown in sticklebacks that repeatability of social tendency may be highest in tanks which have 30 cm between empty and full compartments (McInnes et al. 2025). The position of the 2 shoals was rotated every trial. Individuals used as the social stimulus were always the same species as the focal individual. The temperature of the water in the glass containers was matched to the experimental condition; however, the containers were replaced with 100% DO water between trials and closed to the experimental tanks so that stimulus fish were never exposed to hypoxia.

Two side chambers were separated from the center chamber with solid walls with a pull-door in the center. A model predator (a rubber juvenile rainbow trout) was placed behind the pull-door in one of the side chambers, to be used in the second half of the trial. On either end of the side chambers, there were additional compartments constructed out of mesh that allowed water to pass through, but not the fish. These additional chambers housed the inflow and outflow tubes for the recirculating system that was used to control the level of DO in the experimental arenas. There was an inflow and outflow tube on each side of the arena to facilitate even mixing of the water. The recirculating system worked by pumping water from the arena through a reservoir with either an air stone (100% DO) or a nitrogen bubbler (30% DO) before being returned to the experimental arena. Nitrogen concentration was maintained in the reservoir using a Loligo OXY-REG that activated a solenoid valve connected to a nitrogen tank. When fish were placed in acclimation tubes both arenas were at 100% DO; then one arena (alternating across days) was gradually brought down to 30% DO over the course of ∼10 min. DO in the center of the arena, near the acclimation tubes was monitored using a handheld oxygen monitor during oxygen reduction but removed during the assay. The recirculating system was kept on for the duration of the trial to maintain the appropriate oxygen concentration. Water in the experimental arenas was changed between each trial to control for chemical cues and to ensure that the tanks started at 100% DO.

After 30% DO was reached in one arena, focal fish in both arenas were allowed to acclimate to the experimental conditions for an additional 5 min before the acclimation tube was removed. The focal fish could then move freely around the tank for 17 min. Then, two experimenters manually removed the side doors using a pulley system from behind the curtains such that all doors were removed at the same time. One compartment in each tank was empty; however, a model predator (Fig. 1b) was housed in the other compartment. The model predator was suspended above the center of the tank with transparent fishing line, such that when the door was removed, the predator “swam” (ie, swung) to the center of the tank where it remained until the end of the trial. Fish were allowed to move around the tank for a further 7 min after the release of the predator. All trials were filmed from above using a GoPro 7 and tracked using Ethovision XT 15 (Noldus et al. 2001). All tracks were manually checked and videos were watched at 4× speed for verification. For the “pre-predator” videos, the first 10 min was considered as an acclimation to the arena and only the final 7 min were tracked. Acclimation time was chosen based on manual checks of the videos: initially many fish engaged in erratic exploratory behavior, which had decreased after 10 min. Actual recording was for a minimum of 18 min to ensure a 10-min acclimation period and 7-min tracking period. In addition to measuring overall activity (time spent moving), we also recorded time spent within 1 body length of each of the 4 corner containers (ie, the large and small shoal and the empty controls); time spent within 1 body length of the large shoal was used as the primary measure of sociability. For the “post-predator” release videos, we only considered the minute immediately after the predator was released as many fish responded to the moving model predator with a startle response (ie, as if it was a predator) but after a period in which the predator had not moved, many fish moved under the model predator (ie, treating it as if it was a shelter). All trials were conducted in the same order (ie, behavior was recorded in the absence of the predator prior to the presence of the predator) to minimize carryover effects from the predator that may have influenced subsequent behavior (Bell 2013).

After each trial, the focal fish was euthanized in benzocaine solution (100 mg/L) and total length (minnows = 48.23 ± 7.60 mm, sticklebacks = 47.60 ± 6.49) and mass (minnows = 1.00 ± 0.55 g, sticklebacks = 0.92 ± 0.46 g) were measured to account for body size-related variation in the analyses.

### Statistical analysis

All analyses were performed in R (R version 4.4.0) using the following packages: “tidyverse” (Wickham et al. 2019), “lme4” (Bates et al. 2015), “lmerTest” (Kuznetsova et al. 2017), “emmeans” (Lenth 2025), and “Rmisc” (Hope 2022). To explore whether experimental treatments influenced activity in the absence of predators we fitted a linear mixed model with time spent moving in seconds during the final 7 min of the “pre-predator” trials as the response variable and total length and the interaction between temperature and oxygen level as fixed effects. Trial number within the day was included as a random effect (6 levels). The same model structure with the addition of position of social group as a random effect (4 levels) was used to look at the influence of experimental treatments on sociability prior to predator release. Time spent within 1 body length of the large shoal was used as the response variable. In each case, separate models were run for sticklebacks and minnows. Both temperature and oxygen concentration were modeled as categorical variables with 12 °C and 100% oxygen used as the reference levels in all models. All behavioral data fit the assumptions of a general linear mixed model, and the residuals were normally distributed. An estimated marginal mean for each factor conditional on the other (ie the contrast between high and low temperatures at each oxygen concentration and the contrast between hypoxia and normoxia at each temperature) was calculated using the “emmeans” package.

To increase the power of models and use the entire sample to determine pre-predator differences (ie to not exclude fish where the predator failed to release), we ran pre-predator exposure models separately from the post-predator condition. To analyze the effect of the predator while still accounting of individual differences in behavior prior to the predator release, post-predator models used the same model structure as the pre-predator models except that we included a 3-way interaction between temperature, oxygen, and predator presence/absence. Individual ID was now also included as a random effect to account for the fact that each fish contributed 2 measures for the response variable. Our response variables were still time spent moving and time spent with the group; however, we calculated a 1-min average for each behavior prior to the predator release to make it comparable to the 1-min post-predator data. We calculated the average from the full 7-min data set by dividing the total time spent moving and with the group by 7 to get a representative average. Additionally, we ran the same predator model structures using behavior from the 1-min immediately prior to releasing the predator as opposed to a representative average as the response variables, to assess whether the immediate behavioral state of the fish prior to predator release influenced its response. The results are qualitatively similar between these two approaches and do not substantially influence our interpretation of the data (Tables S1 and S2). As in the pre-predator models, separate models were run for each species, 12 °C and 100% oxygen were used as reference levels for the categorical variables of temperature and hypoxia, and estimated marginal means for each factor conditional on the other were calculated using the “emmeans” package. Calculating and comparing marginal means is useful in interpreting interactions because they are invariant to reference level (Aiken 1991). In discussing our results, we treat *P*-values as a continuous measure of evidence against the null hypothesis as originally described by Fisher (1955) and recently elaborated on by Muff et al. (2022).

### Ethical statement

The experiment was conducted under the approval of the University of Glasgow Animal Welfare and Ethical Review Board and U.K. Home Office project license PP6899400. Care was taken when moving fish between tanks and the experimental arenas to minimize stress. Air exposure was minimal, and we avoided unnecessary chasing of fish throughout the experiment.

## Results

**Figure 2:**
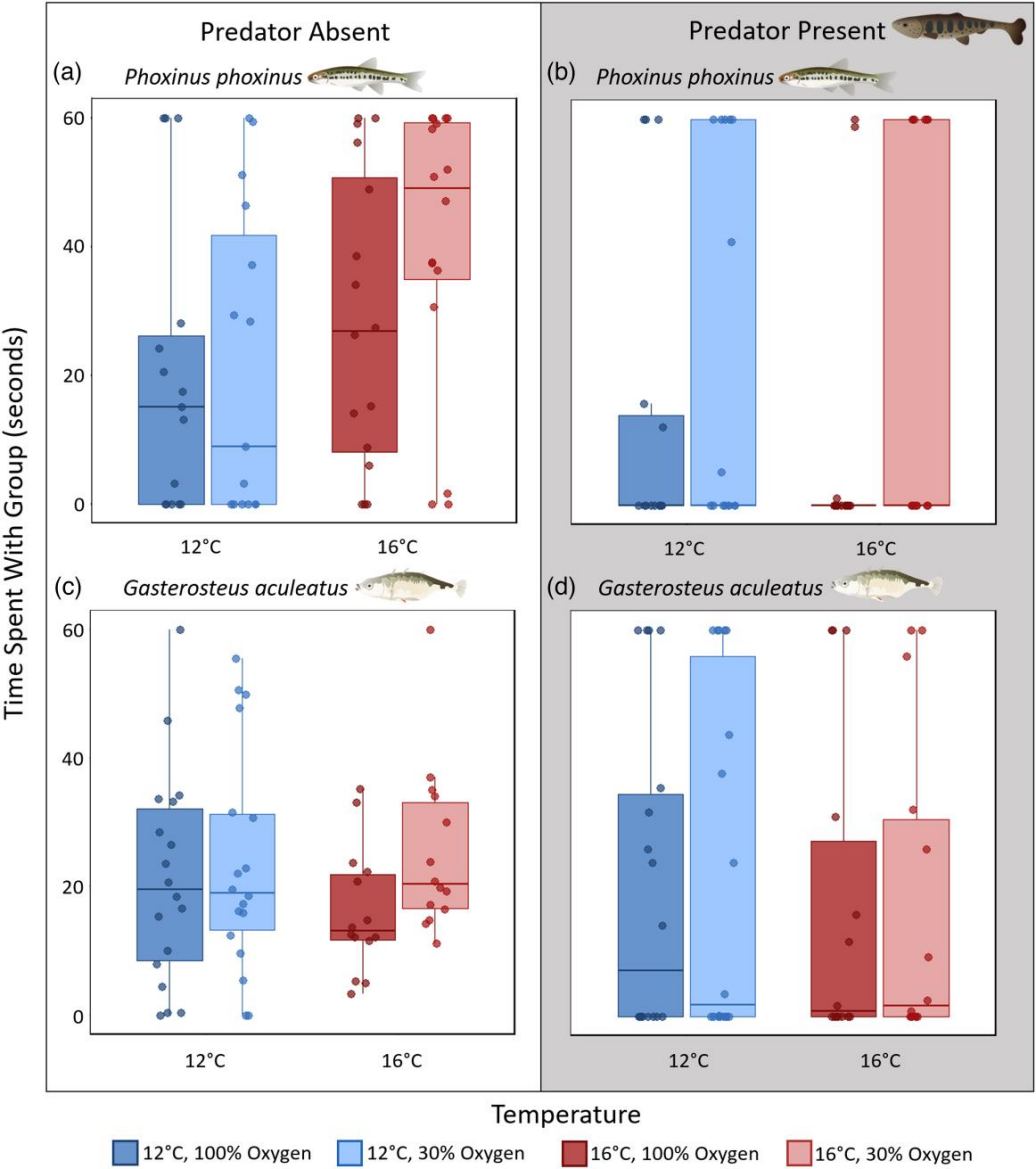
Influence of temperature (12°C: blue bars, 16°C: red bars) and oxygen concentration (100% DO: dark bars, 30% DO: light bars) on time (in seconds) spent with a social group in the absence a, c) and presence b, d) of a model predator in minnows a, b) and sticklebacks c, d). Predator absent data is a 1-min average of a 7-min recording to facilitate comparison between predator absent and present conditions.

Prior to predator release, there was no main effect of temperature or oxygen concentration on time spent with the social group in either minnows or sticklebacks (Table 1, Fig. 2). However, there were trends to suggest that the effect of one stressor depended on the other, ie there were differences between the two temperatures or oxygen levels at the altered level of the other factor. There was weak evidence that minnows spent more time with the social group at 16 °C than at 12 °C under hypoxic conditions (emmeans: −111 ± 59.4, *P* = 0.067) but not at 100% oxygen. Similarly, there was weak evidence that both minnows (emmeans: −86.8 ± 52.3, *P* = 0.103) and, to a lesser extent, sticklebacks (emmeans: −64.3 ± 39.8, *P* = 0.112) spent less time with the social group at 100% oxygen compared with 30% but only at 16 °C. There was no evidence that sticklebacks spent different amounts of time with a group at 12 °C versus 16 °C at either oxygen concentration.

**Table 1:** Linear mixed model structure, outputs and emmeans contrasts of temperature and hypoxia effects on time spent within 1 body length of the social group during 7 min in the absence of a predator.

Model outputs: Minnow							
Fixed effects	Estimate	SE	df	*t*	*P*	m*R*^2^	c*R*^2^
Intercept	7.324	113.152	55.013	0.065	0.949	0.11	0.286
Total Length	2.983	2.182	56.972	1.367	0.177		
Temperature (16 °C)	36.445	56.983	56.764	0.640	0.525		
Oxygen (30%)	12.219	53.991	51.328	0.226	0.822		
Temp (16 °C):Oxygen (30%)	74.601	75.132	51.322	0.993	0.325		

Emmeans Contrast: Minnow Temp12 °C–Temp16 °C							
Oxygen	Estimate	SE	df	*t*	*P*		
100%	−36.4	59.3	56.6	−0.614	0.541		
30%	−111.0	59.4	56.6	−1.869	0.067		

Oxygen (100%)–Oxygen (30%)							
Temperature							
12 °C	−12.2	54.0	49.4	−0.226	0.822		
16 °C	−86.8	52.3	49.4	−1.661	0.103		

Model outputs: Stickleback							
Fixed effects	Estimate	SE	df	*t*	*P*	m*R*^2^	c*R*^2^
Intercept	117.503	129.513	59.0	0.907	0.368	0.047	0.047
Total Length	0.630	2.633	59.0	0.239	0.812		
Temperature (16 °C)	−34.860	37.276	59.0	−0.935	0.354		
Oxygen (30%)	17.796	34.870	59.0	0.510	0.612		
Temp (16 °C):Oxygen (30%)	46.464	52.729	59.0	0.881	0.382		

Emmeans Contrast: Stickleback Temp12 °C–Temp16 °C							
Oxygen	Estimate	SE	df	*t*	*P*		
100%	34.9	38.2	56.8	0.912	0.365		
30%	−11.6	38.7	57.9	−0.30	0.765		

Oxygen (100%)–Oxygen (30%)							
Temperature							
12 °C	−17.8	35.0	53.6	−0.508	0.613		
16 °C	−64.3	39.8	53.8	−1.616	0.112		

Model: Time with Group ∼ Total length + Temperature * Oxygen + (1|Trial) + (1| Group Position).

**Figure 3:**
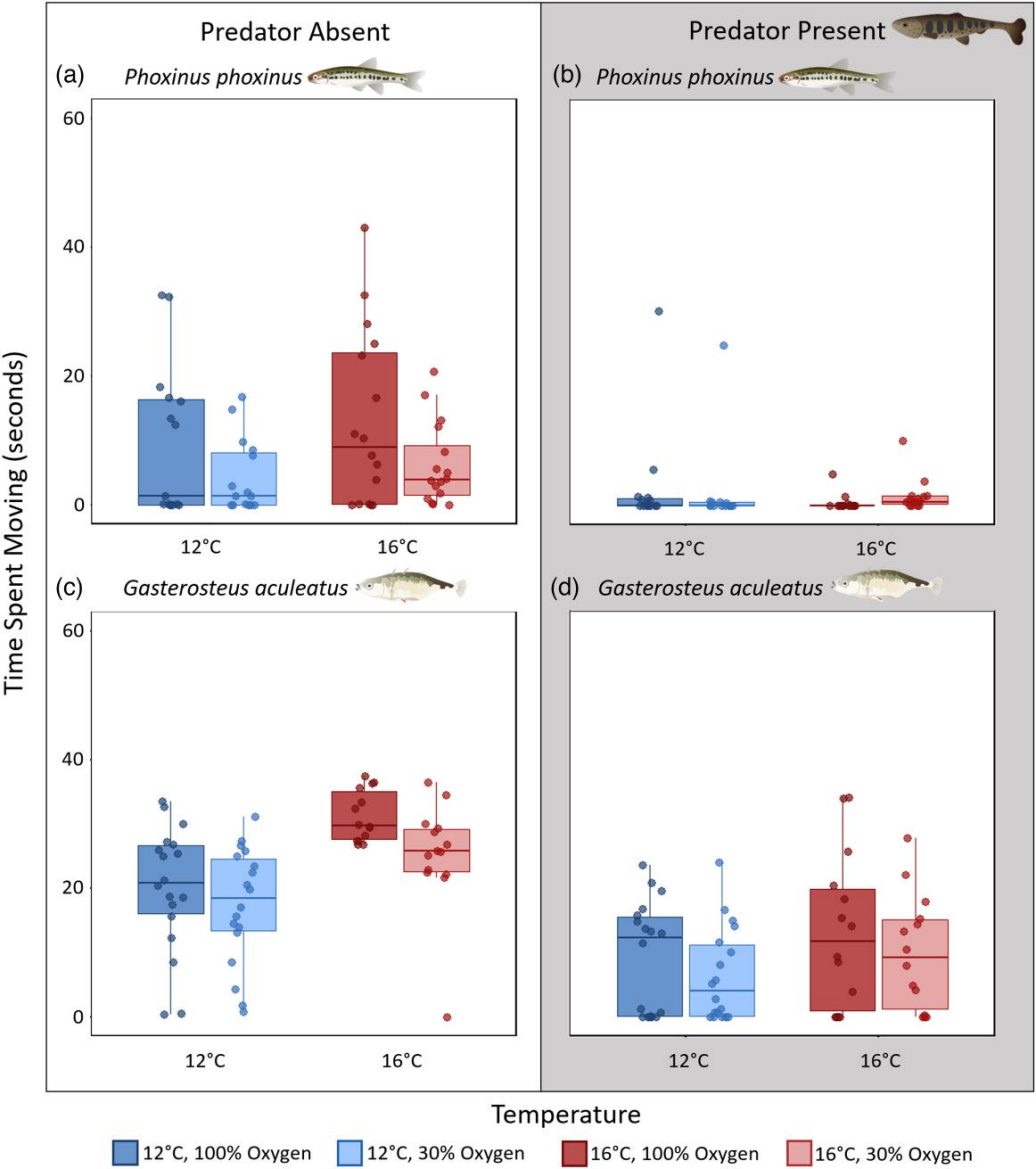
Influence of temperature (12°C: blue bars, 16°C: red bars) and oxygen concentration (100% DO: dark bars, 30% DO: light bars) on time (in seconds) spent moving in the absence a, c) and presence b, d) of a model predator in minnows a, b) and sticklebacks c, d). Predator absent data is a 1-min average of a 7-min recording to facilitate comparison between predator absent and present conditions.

Patterns for the effect of temperature and oxygen concentration on time spent moving differed between sticklebacks and minnows (Table 2, Fig. 3). In minnows, there was a trend to suggest that oxygen concentration had a negative effect on time spent moving (ie fish spent less time moving in hypoxic conditions compared with normoxic conditions) (LMM: −34.856 ± 23.903, *P* = 0.150). This pattern was driven largely by moderate evidence of a decrease in time spent moving in hypoxic conditions at 16 °C (emmeans: 46.9 ± 23.1, *P* = 0.048). Contrary to predictions, temperature did not affect the time that minnows spent moving but there was strong evidence to suggest that it did affect the time that sticklebacks spent moving (LMM: 78.59 ± 20.93, *P* = 0.0004). Sticklebacks moved more at 16 °C than 12 °C at both oxygen concentrations, although the magnitude of the differences was higher at 100% (emmeans: −78.6 ± 21.3, *P* = 0.0005) than 30% oxygen (emmeans: −54.6 ± 21.5, *P* = 0.0137). There was weak evidence of oxygen concentration influencing movement in sticklebacks, such that fish at 100% oxygen moved more than fish at 30% oxygen, but only at 16 °C (emmeans: 42.7 ± 22.3, *P* = 0.06). There was a main effect of total length on activity in minnows (LMM: 2.663 ± 0.881, *P* = 0.003) but not in sticklebacks (LMM: 0.215 ± 1.479, *P* = 0.885).

**Table 2:** Linear mixed model structure, outputs and emmeans contrasts of temperature and hypoxia effects on time spent moving (s) during 7 min in the absence of a predator.

Model outputs: Minnow							
Fixed effects	Estimate	SE	df	*t*	*P*	m*R*^2^	c*R*^2^
Intercept	−60.469	45.359	57	−1.333	0.187	0.217	0.217
Total Length	2.663	0.8806	57	3.024	0.003		
Temperature (16 °C)	19.850	23.562	57	0.842	0.403		
Oxygen (30%)	−34.856	23.903	57	−1.458	0.150		
Temp (16 °C):Oxygen (30%)	−11.997	33.265	57	−0.361	0.719		

Emmeans Contrast: Minnow Temp 12 °C–Temp 16 °C							
Oxygen	Estimate	SE	df	*t*	*P*		
100%	−19.85	24.7	53.4	−0.802	0.426		
30%	−7.85	24.8	53.3	−0.317	0.752		

Oxygen (100%)–Oxygen (30%)							
Temperature							
12 °C	34.9	23.9	51.8	1.458	0.150		
16 °C	46.9	23.1	51.8	2.025	0.048		

Model outputs: Stickleback							
Fixed effects	Estimate	SE	df	*t*	*P*	m*R*^2^	c*R*^2^
Intercept	129.776	72.743	59	1.784	0.079	0.282	0.282
Total Length	0.215	1.479	59	0.146	0.885		
Temperature (16 °C)	78.597	20.937	59	3.754	0.0004		
Oxygen (30%)	−18.712	19.586	59	−0.955	0.343		
Temp (16 °C):Oxygen (30%)	−24.029	29.616	59	−0.811	0.420		

Emmeans Contrast: Stickleback Temp 12 °C–Temp 16 °C							
Oxygen	Estimate	SE	df	*t*	*P*		
100%	−78.6	21.3	57.9	−3.696	0.0005		
30%	−54.6	21.5	58.9	−2.541	0.0137		

Oxygen (100%)–Oxygen (30%)							
Temperature							
12 °C	18.7	19.6	54.9	0.954	0.344		
16 °C	42.7	22.3	55.1	1.919	0.060		

Model: Time Moving ∼ Total length + Temperature * Oxygen + (1|Trial).

There were no strong differences in time spent with a social group before and after the predator was released in either sticklebacks or minnows. Minnows decreased the time they spent with the social group only at 16 °C and 100% oxygen (emmeans: 19.68 ± 5.32, *P* = 0.011) and there was no difference between treatments after the predator was released (Table 3). Sticklebacks did not alter the time spent with the group after the predator was released in any treatment and there were no differences between the treatments after the predator had been released.

**Table 3:** Linear mixed model structure, outputs and emmeans contrasts of temperature, hypoxia and predator presence effects on the time spent within 1 body length of the social group 1 min before (calculated as an average) and after predator release.

Model outputs: Minnow							
Fixed effects	Estimate	SE	df	*t*	*P*	m*R*^2^	c*R*^2^
Intercept	5.422	15.982	57.8	0.339	0.736	0.120	0.708
Total Length	0.341	0.302	56.9	1.128	0.264		
Temperature (16 °C)	4.952	8.721	80.2	0.568	0.572		
Oxygen (30%)	1.694	8.338	76.1	0.203	0.840		
Predator (Present)	−6.239	5.348	56.7	−1.167	0.248		
Temp (16 °C):Oxygen (30%)	10.686	11.603	76.1	0.921	0.360		
Temp (16 °C):Predator (Present)	−13.440	7.541	57.2	−1.782	0.080		
Oxygen (30%):Predator (Present)	7.725	7.564	56.7	1.021	0.311		
Temp (16 °C):Oxygen (30%):Predator (Present)	0.283	10.663	57.2	0.27	0.979		

Selected Emmeans Contrast: Minnow Pred (Absent)–Pred (present)	Estimate	SE	df	*t*	*P*		
Temp (12 °C), Oxygen (100%)	6.240	5.35	56.1	1.167	0.938		
Temp (16 °C), Oxygen (100%)	19.680	5.32	57.2	3.698	0.011		
Temp (12 °C), Oxygen (30%)	−1.486	5.35	56.1	−0.278	1.000		
Temp (16 °C), Oxygen (30%)	11.671	5.32	57.2	2.198	0.371		

Predator Present Contrasts Oxygen (100%)–Oxygen (30%)							
Temperature	Estimate	SE	df	*t*	*P*		
12 °C	−9.419	8.34	73.0	−1.130	0.948		
16 °C	−20.389	8.25	76.4	−2.471	0.224		

Temp (12 °C)–Temp (16 °C)							
Oxygen							
100%	8.488	9.06	80.8	0.937	0.981		
30%	−2.481	9.08	80.9	−0.273	1.000		

Model outputs: Stickleback							
Fixed effects	Estimate	SE	df	*t*	*P*	m*R*^2^	c*R*^2^
Intercept	15.547	20.76	61.5	0.749	0.457	0.019	0.281
Total Length	0.116	0.418	58.9	0.277	0.783		
Temperature (16 °C)	−4.810	7.393	109.0	−0.651	0.517		
Oxygen (30%)	2.323	6.907	108.3	0.336	0.737		
Predator (Present)	−0.496	5.992	60.0	−0.083	0.934		
Temp (16 °C):Oxygen (30%)	7.131	10.447	108.5	0.683	0.496		
Temp (16 °C):Predator (Present)	1.521	9.058	60.0	0.168	0.867		
Oxygen (30%):Predator (Present)	−0.457	8.474	60.0	−0.054	0.957		
Temp (16 °C):Oxygen (30%): Predator (Present)	−8.245	12.811	60.0	−0.644	0.522		

Emmeans Contrast: Stickleback Pred (Absent)–Pred (present)	Estimate	SE	df	*t*	*P*		
Temp (12 °C), Oxygen (100%)	0.496	5.99	60	0.083	1.00
Temp (16 °C), Oxygen (100%)	−1.025	6.79	60	−0.151	1.00
Temp (12 °C), Oxygen (30%)	0.953	5.99	60	0.159	1.00
Temp (16 °C), Oxygen (30%)	7.677	6.79	60	1.130	0.948

Predator Present Contrasts Oxygen (100%)–Oxygen (30%)							
Temperature	Estimate	SE	df	*t*	*P*		
12 °C	−1.866	6.92	103	−0.270	1.00		
16 °C	−0.752	7.85	103	−0.096	1.00		

Temp (12 °C)–Temp (16 °C)							
Oxygen	Estimate	SE	df	*t*	*P*		
100%	3.289	7.51	108	0.438	0.999		
30%	4.403	7.56	109	0.583	0.999		

Model: Time Spent with Group ∼ Total length + Temperature * Oxygen * Predator Presence/Absense + (1|Trial) + (1| Group Position) + (1|ID).

There were, however, changes in the time spent moving after the predator was released. In minnows, there was a trend to suggest a main effect of predator such that fish moved less immediately after the predator had been released compared with before predator release (LMM: −6.845 ± 2.845, *P* = 0.19). This was driven largely by strong evidence of a decrease in the time that minnows spent moving after the predator had been released at 16 °C and 100% (Emmeans: 12.593 ± 2.80, *P* = 0.0008). After the predator was released, there were no differences between treatments in the time that minnows spent moving. There was a strong overall effect of predator on the time that sticklebacks spent moving (LMM: −10.862 ± 2.273, *P ≤* 0.0001) with significant decreases in the time spent moving in all treatment groups (see Table 4) and no differences between treatments after the predator had been released.

**Table 4:** Linear mixed model structure, outputs and emmeans contrasts of temperature, hypoxia and predator presence effects on the time spent moving 1 min before (calculated as an average) and after predator release.

Model outputs: minnow							
Fixed effects	Estimate	SE	df	*t*	*P*	m*R*^2^	c*R*^2^
Intercept	0.602	4.116	70.4	0.146	0.884	0.250	0.256
Total Length	0.187	0.075	55.4	2.495	0.015		
Temperature (16 °C)	3.142	2.815	112.9	1.116	0.267		
Oxygen (30%)	−5.097	2.858	112.9	−1.783	0.077		
Predator (Present)	−6.845	2.845	56.1	−2.405	0.019		
Temp (16 °C):Oxygen (30%)	−1.649	3.978	112.9	−0.415	0.679		
Temp (16 °C):Predator (Present)	−5.748	3.993	60.0	−1.439	0.155		
Oxygen (30%):Predator (Present)	4.318	4.024	56.1	1.073	0.288		
Temp (16 °C):Oxygen (30%): Predator (Present)	3.482	5.648	60.0	0.616	0.540		

Emmeans Contrast: Minnow Pred (Absent)–Pred (present)	Estimate	SE	df	*t*	*P*		
Temp (12 °C), Oxygen (100%)	6.846	2.85	56.5	2.405	0.259		
Temp (16 °C), Oxygen (100%)	12.593	2.80	58.4	4.491	0.0008		
Temp (12 °C), Oxygen (30%)	2.527	2.85	56.5	0.888	0.986		
Temp (16 °C), Oxygen (30%)	4.793	2.80	58.4	1.709	0.681		

Predator Present Contrasts Oxygen (100%)–Oxygen (30%)							
Temperature	Estimate	SE	df	*t*	*P*		
12 °C	0.779	2.86	107.3	0.273	1.000		
16 °C	−1.054	2.86	107.3	−0.368	1.000		

Temp (12 °C)–Temp (16 °C)							
Oxygen	Estimate	SE	df	*t*	*P*		
100%	2.606	2.93	110.5	0.889	0.986		
30%	0.773	2.94	110.3	0.263	1.000		

Model outputs: Stickleback							
Fixed effects	Estimate	SE	df	*t*	*P*	m*R*^2^	c*R*^2^
Intercept	12.950	9.158	59.5	1.414	0.163	0.446	0.670
Total Length	0.146	0.184	56.9	0.794	0.430		
Temperature (16 °C)	11.295	3.131	100.4	3.607	0.0005		
Oxygen (30%)	−2.677	2.920	97.6	−0.916	0.362		
Predator (Present)	−10.862	2.273	60.0	−4.779	<0.0001		
Temp (16 °C):Oxygen (30%)	−3.446	4.416	97.7	−0.780	0.437		
Temp (16 °C):Predator (Present)	−7.276	3.436	60.0	−2.118	0.038		
Oxygen (30%):Predator (Present)	−0.078	3.214	60.0	−0.025	0.981		
Temp (16 °C):Oxygen (30%): Predator (Present)	2.960	4.859	60.0	0.609	0.545		

Emmeans Contrast: Stickleback Pred (absent)–Pred (present)	Estimate	SE	df	*t*	*P*		
Temp (12 °C), Oxygen (100%)	10.862	2.27	60.0	4.779	0.0003
Temp (16 °C), Oxygen (100%)	18.139	2.58	60.0	7.039	<0.0001
Temp (12 °C), Oxygen (30%)	10.941	2.27	60.0	4.814	0.0003
Temp (16 °C), Oxygen (30%)	15.259	2.58	60.0	5.921	<0.0001

Predator Present Contrasts Oxygen (100%)–Oxygen (30%)							
Temperature	Estimate	SE	df	*t*	*P*		
12 °C	2.756	2.92	96.1	0.942	0.981		
16 °C	3.242	3.32	96.3	0.977	0.976		

Temp (12 °C)–Temp (16 °C)							
Oxygen	Estimate	SE	df	*t*	*P*		
100%	−4.018	3.16	99.9	−1.271	0.907		
30%	−3.532	3.19	101.5	−1.107	0.954		

Model: Time Moving ∼ Total length + Temperature * Oxygen * Predator + (1|Trial) + (1|ID).

## Discussion

Acute exposures to elevated temperature and decreased oxygen concentration had differing effects on activity and social behavior in the two fish species we examined, despite these species frequently co-occurring in the same habitats and occupying similar ecological niches. For example, sticklebacks showed a general increase in activity with temperature whereas there was weak evidence to suggest that social behavior and activity in minnows was only affected by the combination of factors. Following the release of a model predator, there were no differences among treatment groups in either species, due, in part, to general reductions in activity.

Taken together, these results underscore the importance of considering multiple stressors and interactions between environmental effects when trying to predict organismal behavioral responses. Exposure to multiple stressors have the potential to have synergistic effects that are greater than the sum of the effects of the individual stressors (Todgham and Stillman 2013), particularly if exposure to one stressor exacerbates the effect of the other. While sticklebacksincreased the time they spent moving in warm water, generally changes in behavior were only apparent, or were stronger, in response to one stressor conditional on the other factor, particularly for minnows. For example, there was a trend to suggest that minnows altered their behavior the most in warm hypoxic water: they reduced their activity relative to normoxic warm water and spent more time with the group relative to cool hypoxic water. It is perhaps not surprising that the effect of decreased oxygen is more apparent at higher temperatures. In ectotherms, elevated temperatures can increase the efficiency and speed of a variety of metabolic processes (Schulte 2015); however, many of these processes require oxygen. In warm hypoxic conditions, fish may thus have to alter their behavior to obtain enough oxygen to meet their increased metabolic needs (McBryan et al. 2013). Elevated temperature may only have minimal effects on behavior when there is sufficient oxygen available, and the influence of low oxygen may not be apparent when the individual does not require additional oxygen.

Following the release of the model predator, there were no differences between the treatment groups in either of our behavior measures. All fish appeared to respond strongly to the predator by freezing immediately after release. While there was only strong evidence for a main effect of predator presence on time spent moving for sticklebacks, this may be due to higher moving times prior to the predator release in sticklebacks relative to minnows. Although minnows essentially did not move after the predator was released, the magnitude of their change in activity was smaller. Importantly, this general reduction in time spent moving means that interpreting patterns of social behavior is challenging. Particularly for minnows, following the release of the model predator, individuals either spent all of their time with the group or none of it. From watching the videos manually during tracking verification, many fish froze in place when the predator was released and did not move either toward or away from the group. This suggests that in the minute after a predator becomes visible, fish prioritize not drawing attention to themselves over moving to a group, even if schooling may offer protection from future predators. Generally, this suggests that animals may prioritize immediate dangers when responding to multiple conflicting environmental conditions; the need to respond to the predator reduces behavioral variation present from temperature and oxygen concentration in the absence of the predator.

Changes in behavior are not trivial and can have impacts on fitness. Sticklebacks, for example, were more active in warmer water–increased activity could increase the need to forage and put fish at a greater risk of predation (Brown and Kotler 2004; Killen and Brown 2006; Killen et al. 2024a). While in our study, all fish tended to reduce activity following a model predator “attack,” fish that are more active may be more at risk of interacting with predators as they move through the environment or may be less attentive to predators, particularly in more complex environments (Quadros et al. 2019). Additionally, if fish need to be more active in warm water to locate additional food and compensate for increased energy demands, then the reduction in activity following predator release may be more costly in warm water relative to cool water. There was weak evidence to suggest that minnows were more social and less active in warm hypoxic water relative to warm normoxic water. Reducing activity could reduce oxygen uptake and, in this experiment, fish that were less active were more likely to settle near the group. Staying close to the group may also be beneficial for fish that are conserving energy, as swimming in a group can be less energetically costly (Marras et al. 2015; Killen et al. 2024b). While fish that reduce their activity may be less likely to come across predators as they move through the environment, if their movement is constrained by oxygen availability and they cannot swim as fast, they may be more likely to be caught by predators that do find them (Domenici et al. 2007). Having the group's “many eyes” available for predator detection may thus be beneficial to minnows in warm hypoxic waters (Lima 1995). Considering the ecological consequences of changes in behavior in response to environmental conditions may help to explain patterns of survival in wild populations.

Perhaps the most striking pattern in our results is that minnows and sticklebacks differed in their responses to environmental factors, despite being captured at the same time and location and filling similar ecological niches. This could be due to physiological differences between the two species such that the changes in temperature and oxygen differed in how stressful they were to individuals of each species; population-specific tests of the critical thermal maximum (CT_max_) and oxygen tension (*P*_crit_) could be used to determine where in relation to this value the changes to temperature and oxygen in this experiment fell. However, their responses could also reflect different strategies for responding to changes in the environment. Both minnows and sticklebacks are widespread across a range of different environmental conditions, but their wide geographical ranges may have been achieved with different strategies, specifically with regard to investment in anti-predator morphology. Predator threat may change over different environmental conditions if, for example, metabolic properties give predators an advantage over prey at warmer temperatures (McInturf et al. 2022). Sticklebacks are heavily armored and the investment in spines may allow them to alter their behavior less in changing environmental conditions if the dangers associated with predators do not change with the environment for them, as they may for the less protected minnow (Mathis and Chivers 2003). Minnows may rely more on social behavior and activity modulation for safety, so, compared with sticklebacks, they may have to alter behavior more to compensate for potential increased risk of predation as environmental parameters change. Generally, stickleback, which, here, increased their activity in warm water, may be prioritizing maintaining competitive ability and access to resources even under changed conditions. On the other hand, there was a trend to suggest that minnows reduced activity and increased social behavior in the most stressful treatment (ie, elevated temperature and low oxygen). This may represent an energy-conservation strategy that helps them to withstand periods of environmental change.

Interspecific variation in response to environmental stress may constitute an important, understudied, aspect of ecology, particularly for co-occurring species that interact. While there is an interest in differential responses to environmental change between predators and prey (eg McInturf et al. 2022), there is less of a focus on the responses of species that interact in other ways. For example, many species, particularly those that share common predators, may benefit from grouping together (Paijmans et al. 2019). Mixed species social groups have been documented in a variety of animals (eg birds (Morse 1970), mammals (Stensland et al. 2003), and fish (Krause and Godin 1994; Hoare et al. 2000a)). While some benefits of grouping are reduced in mixed-species groups (eg, access to mates), others, including predator detection and avoidance, may be only minimally affected or even improved through the use of heterospecific cues in mixed shoals (Wolters and Zuberbühler 2003; Lukas et al. 2023). Mixed-species groups may also experience reduced competition for some resources if there is more niche partitioning between species than between individuals of the same species (Morse 1970; Kudavidanage et al. 2007). The spread of social information between species in mixed assemblages may also increase foraging opportunities (Webster et al. 2008). Both minnows (Allan and Pitcher 1986; Ward et al. 2003) and sticklebacks (Hoare et al. 2000b; Webster et al. 2008; Ward et al. 2018) have been found to shoal with other species. As they share many of the same predators, where they co-occur, as they do in our system, they are likely to shoal together. However, if species differ in their responses to environmental changes, historic relationships between species may change as a consequence. The different responses that we saw between minnows and sticklebacks could lead to reduced cohesion and effectiveness of mixed-species shoals in changing environments. This could exacerbate the effect of predators if the effective group size decreases because of diverging responses between species. Dedicated studies looking at attraction to heterospecifics and willingness to compromise preferred swim speed (Herbert-Read et al. 2013) in mixed groups under different environmental conditions are needed to better understand community-level impacts of environmental change. However, prolonged or more frequent or extreme changes in conditions may lead to changes in species distributions (Dahms and Killen 2023) such that, in some cases, previously interacting species no longer co-occur, further impacting community dynamics (Hellmann et al. 2012).

Interestingly, there was no effect of body length on social behavior, and body length only influenced activity in minnows, not sticklebacks. Because there is significant size-dependent predator threat in fish, we predicted that smaller fish should be more social than larger fish (Hoare et al. 2000a); we saw no evidence of this here. Similarly, the pace-of-life hypothesis proposes that individuals with a high growth rate should also be more active (Réale et al. 2010). While larger minnows were more active, the same relationship was not found in sticklebacks. This could reflect differences in how predator threat scales with body size between the two species. Heavily armored stickleback may experience less variation in predator threat between size classes compared with minnows. Evidence for the predicted relationship between growth rate and activity in wild fish populations is mixed (eg, Rennie et al. 2005; Biro and Stamps 2008; Laskowski et al. 2016) prompting further consideration of what ecological factors could drive correlations between size and activity.

Although both acute and chronic changes in temperature and oxygen are likely in the lives of fish, time scale of the change could alter the effect. Here, we looked at the effects of acute changes. Chronic exposure to an environmental condition can result in acclimation. For example, sticklebacks exhibited neuroendocrine responses to acute hypoxia (20% DO) that were not apparent after prolonged exposure (O’Connor et al. 2011). However, past experience also mattered: fish from the population that was least likely to experience hypoxia also showed the biggest effects of acute exposure (O’Connor et al. 2011). Elevated temperature similarly has different effects on behavior and physiology depending on the intensity and duration of exposure (Grigaltchik et al. 2012; Alfonso et al. 2021). Chronic exposure to elevated temperature and decreased oxygen availability are likely to have different effects on activity and social behavior than what we found here.

The results here demonstrate that predicting organismal responses to environmental change requires understanding not only how multiple stressors interact, but also how different species respond to these interactions. When considering the effect of temperature and hypoxia on behavior, the presence of more than one stressor appears to be important for revealing changes in behavior. Particularly in the case of minnows, there was only evidence to suggest that behavior was impacted when both stressors were experienced together. However, the addition of a third stressor, the presence of a predator, masked differences in behavior that were present prior to predator release. This illustrates how multiple stressors may alter behavior in ways that are not predictable from responses to a single stressor (Todgham and Stillman 2013; Munson et al. 2024). Animals may be able to compensate for a change in one factor but be unable to do so when multiple stressors are present simultaneously. This suggests that timing is particularly important: we only considered the immediate response to the predator. A longer observation of behavior may reveal different patterns in how stressors are prioritized. Alternatively, the impact of one stressor may always supersede responses to other stressors, such that it masks variation. These results highlight the complexity of predicting ecosystem responses to environmental change and emphasize the importance of considering both multiple stressors and multiple species when evaluating the impacts of warming and deoxygenation on aquatic communities. As climate change continues to alter aquatic environments, understanding these species-specific responses to interacting stressors will be crucial for predicting and managing changes in community structure and ecosystem function.

## Supplementary Material

arag083_Supplementary_Data

## Data Availability

Analyses reported in this article can be reproduced using the data provided by Munson et al. (2026).
