# Supplementary material for: Effects of multiple stressors on the behavior of fish in the presence and absence of a predator
Source: Behav Ecol. 2026 Jul 29;37(5):arag083. doi: 10.1093/beheco/arag083 (PMC13448412; doi:10.1093/beheco/arag083)
Supplement: Supplementary file 1 [file arag083_supplementary_data.docx]

**Supplemental Table 1**

Linear mixed model structure, outputs and emmeans contrasts of temperature, hypoxia and predator presence effects on the time spent within one body length of the social group one minute immediately before and after predator release.

| **Model outputs: Minnow** | | | | | | | | |
| --- | --- | --- | --- | --- | --- | --- | --- | --- |
| Fixed effects | Estimate | | SE | *df* | *t* | *p* | m*R*^2^ | c*R*^2^ |
| Intercept | 9.124 | | 16.729 | 59.5 | 0.545 | 0.588 | 0.113 | 0.561 |
| Total Length | 0.209 | | 0.315 | 54.0 | 0.662 | 0.511 |  |  |
| Temperature(16°C) | 6.990 | | 9.602 | 91.4 | 0.728 | 0.469 |  |  |
| Oxygen(30%) | 2.178 | | 9.286 | 85.6 | 0.235 | 0.815 |  |  |
| Predator(Present) | -4.200 | | 7.032 | 56.6 | -0.597 | 0.553 |  |  |
| Temp(16°C):Oxygen(30%) | 13.868 | | 12.923 | 85.6 | 1.073 | 0.286 |  |  |
| Temp(16°C):Predator(Present) | -14.006 | | 9.902 | 57.3 | -1.414 | 0.163 |  |  |
| Oxygen(30%):Predator(Present) | 7.160 | | 9.945 | 56.6 | 0.720 | 0.475 |  |  |
| Temp(16°C ) :Oxygen(30%):  Predator(Present) | -3.625 | | 14.001 | 57.3 | -0.259 | 0.797 |  |  |
| **Selected Emmeans Contrast: Pred (Absent)-Pred (present)** | **Minnow** | |  |  |  |  |  |  |
|  | Estimate | | SE | *df* | *t* | *p* |  |  |
| Temp(12°C), Oxygen(100%) | 4.20 | | 7.03 | 56.1 | 0.597 | 0.999 |  |  |
| Temp(16°C), Oxygen(100%) | 18.21 | | 6.98 | 57.6 | 2.608 | 0.174 |  |  |
| Temp(12°C), Oxygen(30%) | -2.96 | | 7.03 | 56.1 | -0.421 | 0.999 |  |  |
| Temp(16°C), Oxygen(30%) | 14.67 | | 6.98 | 57.6 | 2.101 | 0.426 |  |  |
| **Predator Present Contrasts**  **Oxygen(100%)- Oxygen(30%)** |  | |  |  |  |  |  |  |
| Temperature | Estimate | | SE | *df* | *t* | *p* |  |  |
| 12°C | -9.34 | | 9.29 | 84.0 | -1.006 | 0.972 |  |  |
| 16°C | -19.58 | | 9.24 | 87.0 | -2.120 | 0.411 |  |  |
| **Temp(12°C) – Temp(16°C)** |  | |  |  |  |  |  |  |
| Oxygen | Estimate | | SE | *df* | *t* | p |  |  |
| 100% | 7.02 | | 10.00 | 92.4 | 0.701 | 0.997 |  |  |
| 30% | -3.23 | | 10.00 | 92.4 | -0.322 | 1.000 |  |  |
| **Model outputs: Sticklebacks** | | | | | | | | |
| Fixed effects | Estimate | | SE | *df* | *t* | *p* | m*R*^2^ | c*R*^2^ |
| Intercept | 14.788 | | 22.101 | 58.2 | 0.669 | 0.506 | 0.032 | 0.181 |
| Total Length | 0.178 | | 0.443 | 53.7 | 0.402 | 0.689 |  |  |
| Temperature(16°C) | -12.975 | | 8.270 | 113.9 | -1.569 | 0.119 |  |  |
| Oxygen(30%) | -5.408 | | 7.708 | 110.4 | -0.702 | 0.484 |  |  |
| Predator(Present) | -2.719 | | 7.200 | 60.0 | -0.378 | 0.707 |  |  |
| Temp(16°C):Oxygen(30%) | 20.181 | | 11.658 | 110.4 | 1.731 | 0.086 |  |  |
| Temp(16°C):Predator(Present) | 9.488 | | 10.889 | 60.0 | 0.872 | 0.387 |  |  |
| Oxygen(30%):Predator(Present) | 7.139 | | 10.183 | 60.0 | 0.701 | 0.486 |  |  |
| Temp(16°C ) :Oxygen(30%):  Predator(Present) | -21.329 | | 15.395 | 60.0 | -1.385 | 0.171 |  |  |
| **Emmeans Contrast: Stickleback**  **Pred(Absent)-Pred(present)** | |  |  |  |  |  |  |  |
|  | Estimate | | SE | *df* | *t* | *p* |  |  |
| Temp(12°C), Oxygen(100%) | 2.719 | | 7.20 | 60 | 0.378 | 0.999 |  |  |
| Temp(16°C), Oxygen(100%) | -6.769 | | 8.16 | 60 | -0.829 | 0.991 |  |  |
| Temp(12°C), Oxygen(30%) | -4.420 | | 7.20 | 60 | -0.614 | 0.999 |  |  |
| Temp(16°C), Oxygen(30%) | 7.421 | | 8.16 | 60 | 0.909 | 0.984 |  |  |
| **Predator Present Contrasts**  **Oxygen(100%)- Oxygen(30%)** |  | |  |  |  |  |  |  |
| Temperature | Estimate | | SE | *df* | *t* | *p* |  |  |
| 12°C | -1.731 | | 7.72 | 109 | -0.224 | 1.000 |  |  |
| 16°C | -0.582 | | 8.76 | 109 | -0.066 | 1.000 |  |  |
| **Temp(12°C) – Temp(16°C)** |  | |  |  |  |  |  |  |
| Oxygen | Estimate | | SE | *df* | *t* | *p* |  |  |
| 100% | 3.488 | | 8.37 | 113 | 0.417 | 0.999 |  |  |
| 30% | 4.636 | | 8.43 | 114 | 0.550 | 0.999 |  |  |

Model: Time Spent with Group ~ Total length + Temperature*Oxygen*Predator Presence/Absense + (1|Trial) + (1| Group Position) + (1|ID)

**Supplemental Table 2**

Linear mixed model structure, outputs and emmeans contrasts of temperature, hypoxia and predator presence effects on the time spent moving one minute immediately before and after predator release.

| **Model outputs: Minnow** | | | | | | | | |
| --- | --- | --- | --- | --- | --- | --- | --- | --- |
| Fixed effects | Estimate | | SE | *df* | *t* | *p* | m*R*^2^ | c*R*^2^ |
| Intercept | 0.383 | | 4.964 | 86.7 | 0.077 | 0.939 | 0.155 | 0.169 |
| Total Length | 0.178 | | 0.091 | 77.9 | 1.962 | 0.053 |  |  |
| Temperature(16°C) | 1.848 | | 3.362 | 111.2 | 0.550 | 0.584 |  |  |
| Oxygen(30%) | -5.755 | | 3.377 | 107.7 | -1.704 | 0.091 |  |  |
| Predator(Present) | -6.093 | | 3.377 | 107.7 | -1.804 | 0.074 |  |  |
| Temp(16°C):Oxygen(30%) | -0.373 | | 4.701 | 107.7 | -0.079 | 0.937 |  |  |
| Temp(16°C):Predator(Present) | -4.758 | | 4.739 | 107.8 | -1.004 | 0.318 |  |  |
| Oxygen(30%):Predator(Present) | 4.971 | | 4.776 | 107.7 | 1.041 | 0.300 |  |  |
| Temp(16°C ) :Oxygen(30%):  Predator(Present) | 2.220 | | 6.701 | 107.7 | 0.331 | 0.741 |  |  |
| **Emmeans Contrast: Minnow**  **Pred(Absent)-Pred(present)** |  | |  |  |  |  |  |  |
|  | Estimate | | SE | *df* | *t* | *p* |  |  |
| Temp(12°C), Oxygen(100%) | 6.093 | | 3.38 | 56.5 | 1.804 | 0.620 |  |  |
| Temp(16°C), Oxygen(100%) | 10.851 | | 3.33 | 58.4 | 3.261 | 0.037 |  |  |
| Temp(12°C), Oxygen(30%) | 1.123 | | 3.38 | 56.5 | 0.331 | 1.000 |  |  |
| Temp(16°C), Oxygen(30%) | 3.661 | | 3.33 | 58.4 | 1.100 | 0.954 |  |  |
| **Predator Present Contrasts**  **Oxygen(100%)-Oxygen(30%)** |  | |  |  |  |  |  |  |
| Temperature | Estimate | | SE | *df* | *t* | *p* |  |  |
| 12°C | 0.785 | | 3.38 | 107.3 | 0.232 | 1.000 |  |  |
| 16°C | -1.062 | | 3.38 | 107.3 | -0.314 | 1.000 |  |  |
| **Temp(12°C) – Temp(16°C)** |  | |  |  |  |  |  |  |
| Oxygen | Estimate | | SE | *df* | *t* | *p* |  |  |
| 100% | 2.910 | | 3.49 | 112.0 | 0.834 | 0.991 |  |  |
| 30% | 1.063 | | 3.49 | 111.9 | 0.304 | 1.000 |  |  |
| **Model outputs: Sticklebacks** | | | | | | | | |
| Fixed effects | Estimate | | SE | *df* | *t* | *p* | m*R*^2^ | c*R*^2^ |
| Intercept | 11.770 | | 10.837 | 59.1 | 1.086 | 0.282 | 0.372 | 0.683 |
| Total Length | 0.121 | | 0.219 | 57.1 | 0.553 | 0.582 |  |  |
| Temperature(16°C) | 14.037 | | 3.588 | 93.7 | 3.913 | 0.0002 |  |  |
| Oxygen(30%) | -0.273 | | 3.354 | 90.6 | -0.081 | 0.935 |  |  |
| Predator(Present) | -8.459 | | 2.387 | 60.0 | -3.544 | 0.0008 |  |  |
| Temp(16°C):Oxygen(30%) | -7.059 | | 5.071 | 90.7 | -1.392 | 0.167 |  |  |
| Temp(16°C):Predator(Present) | -10.064 | | 3.609 | 60.0 | -2.789 | 0.007 |  |  |
| Oxygen(30%):Predator(Present) | -2.493 | | 3.376 | 60.0 | -0.739 | 0.463 |  |  |
| Temp(16°C ) :Oxygen(30%):  Predator(Present) | 6.615 | | 5.103 | 60.0 | 1.296 | 0.200 |  |  |
| **Emmeans Contrast: Stickleback**  **Pred(Absent)-Pred(present)** | |  |  |  |  |  |  |  |
|  | Estimate | | SE | *df* | *t* | *p* |  |  |
| Temp(12°C), Oxygen(100%) | 8.459 | | 2.39 | 60.0 | 3.544 | 0.017 |  |  |
| Temp(16°C), Oxygen(100%) | 18.523 | | 2.71 | 60.0 | 6.844 | <0.0001 |  |  |
| Temp(12°C), Oxygen(30%) | 10.952 | | 2.39 | 60.0 | 4.588 | 0.0006 |  |  |
| Temp(16°C), Oxygen(30%) | 14.401 | | 2.71 | 60.0 | 5.321 | <0.0001 |  |  |
| **Predator Present Contrasts**  **Oxygen(100%)-Oxygen(30%)** |  | |  |  |  |  |  |  |
| Temperature | Estimate | | SE | *df* | *t* | *p* |  |  |
| 12°C | 2.766 | | 3.36 | 89.1 | 0.824 | 0.991 |  |  |
| 16°C | 3.211 | | 3.81 | 89.3 | 0.843 | 0.990 |  |  |
| **Temp(12°C) – Temp(16°C)** |  | |  |  |  |  |  |  |
| Oxygen | Estimate | | SE | *df* | *t* | *p* |  |  |
| 100% | -3.973 | | 3.63 | 93.3 | -1.095 | 0.956 |  |  |
| 30% | -3.528 | | 3.66 | 94.8 | -0.965 | 0.978 |  |  |

Model : Time Moving ~ Total length + Temperature*Oxygen*Predator + (1|Trial) + (1|ID)
